# The Utility of Rural and Underserved Designations in Geospatial Assessments of Distance Traveled to Healthcare Services: Implications for Public Health Research and Practice

**DOI:** 10.1155/2013/960157

**Published:** 2013-06-13

**Authors:** Matthew Lee Smith, Justin B. Dickerson, Monica L. Wendel, SangNam Ahn, Jairus C. Pulczinski, Kelly N. Drake, Marcia G. Ory

**Affiliations:** ^1^The University of Georgia, College of Public Health, Department of Health Promotion and Behavior, 330 River Road, 315 Ramsey Center, Athens, GA 30602, USA; ^2^Texas A&M Health Science Center, School of Rural Public Health, Department of Health Promotion and Community Health Sciences, TAMU 1266, College Station, TX 77843, USA; ^3^HSR Health Services, LLC, P.O. Box 571357, Murray, UT 84157, USA; ^4^Texas A&M Health Science Center, School of Rural Public Health, Department of Health Policy and Management, TAMU 1266, College Station, TX 77843, USA; ^5^The University of Memphis, School of Public Health, Division of Health Systems Management and Policy, Robison Hall 133, Memphis, TN 38152-3530, USA; ^6^Texas A&M Health Science Center, School of Rural Public Health, Center for Community Health Development, TAMU 1266, College Station, TX 77843, USA

## Abstract

Health disparities research in rural populations is based on several common taxonomies identified by geography and population density. However, little is known about the implications of different rurality definitions on public health outcomes. To help illuminate the meaning of different rural designations often used in research, service delivery, or policy reports, this study will (1) review the different definitions of rurality and their purposes; (2) identify the overlap of various rural designations in an eight-county Brazos Valley region in Central Texas; (3) describe participant characteristic profiles based on distances traveled to obtain healthcare services; and (4) examine common profile characteristics associated with each designation. Data were analyzed from a random sample from 1,958 Texas adults participating in a community assessment. K-means cluster analysis was used to identify natural groupings of individuals based on distance traveled to obtain three healthcare services: medical care, dental care, and prescription medication pick-up. Significant variation in cluster representation and resident characteristics was observed by rural designation. Given widely used taxonomies for designating areas as rural (or provider shortage) in health-related research, this study highlights differences that could influence research results and subsequent program and policy development based on rural designation.

## 1. Introduction

According to the United States Census, in 2010, there were approximately 60 million adults or 20% of the population living in rural areas [1]. Rural areas have been characterized by well-documented health disparities in terms of healthcare access and health outcomes [2–5]. Distance to health care is often attributed as a barrier to obtaining needed healthcare [6]. Additionally, residents of rural communities are more likely to have higher rates of poverty, poorer educational attainment, and unique health issues such as morbidity and mortality from agriculture, mining, forestry, and fishing [4]. Given the diversity of rural settings within and across different states and regional areas, it is difficult to generalize health risks and access to care in these diverse populations.

Public health researchers, practitioners, and policy-makers often use underserved and rural interchangeably when referring to areas with low population density, but to what extent do these two terms carry similar meaning? Our current understanding of rurality and rural designations is based largely on research using one of several common taxonomies related to geography, population density, and distance to certain functions such as employment, food, and health services. While the existing research is informative, it is critical to acknowledge that the rural designations used were not developed specifically for public health research, understanding health disparities, and informing health policy, although they are routinely applied this way. Thus, these designations may not capture important aspects of rurality when applied this way. Defining “rural” for health research and policy development demands that we specify which dimensions of rurality are most relevant to a specific issue and select a taxonomy accordingly. Existing definitions for categorizing “rural” and “urban” often disregard significant cultural, demographic, and socioeconomic characteristics that have critical implications for health disparities research and policy [7].

Thus, the general purposes of this study were to review the different definitions of rurality and their purposes and examine common resident profile characteristics associated with each designation using an eight-county sample from Central Texas. Such an examination will add to the research about the consistency between and application of taxonomies used to define areas as rural or having a provider shortage, which has implications for research results and subsequent program and policy development based on rural designation (see specific study aims description later).

## 2. Definitions of Rural

As indicated by the Office of Rural Health Policy [8] there are two overarching US designations that are used: a Census Bureau definition and an Office of Management and Budget (OMB) definition. The Census Bureau uses population density and population clusters to define urban regions; everything that does not constitute an urban area is defined as “nonmetropolitan” or rural. The OMB defines counties as metropolitan or nonmetropolitan based on the population of cities within counties and the counties' proximity to other metropolitan counties. This classification is important because it provides the framework for many other designations. A metropolitan area can exist across multiple counties, and as long as 50% of the county falls under the metropolitan designation, the entire county is considered metropolitan [5, 9].

The definition of nonmetropolitan applied by the National Center for Health Statistics largely follows the Census and OMB, as it deals with much of the same data, dividing nonmetropolitan counties into “micropolitan” and “noncore” [9]. In addition to the Census Bureau and OMB definitions, the US Department of Agriculture (USDA) devised Urban Influence Codes (UICs) to stratify the OMB county definitions on a 12-point scale. The scale accounts for city size and commuting characteristics but still uses counties as the unit of measurement [10–12]. The USDA also developed Rural Urban Continuum Codes (RUCC), using a 9-point scale that distributes counties into three metropolitan and six nonmetropolitan rankings also accounting for commuting and proximity to a nearby metro area [10, 11]. Rural Urban Commuting Area (RUCA) codes are similar to the Rural Urban Continuum Codes, but they also incorporate commuting data from census tracts into their criteria. Additionally, distance to urban areas or clusters were added to the most recent version of Rural Urban Commuting Area codes, using zip codes to measure the approximate distance to the urban regions [10, 11]. A separate measure exists to calculate extremely rural—or frontier area—regions, based on a matrix that calculates population density and the distance and time it takes to reach services [13].

Each of the current rural definitions was developed for a specific purpose; when used in other applications, each has its limitations. The Census Bureau definition is oversimplified, counting any area rural that is not urban. This false dichotomy misses a great deal of variation in important community characteristics outside large metro areas. The OMB definition and all the codes that use it as a basis for their stratification have the issue of over- and underbounding, from the use of counties as statistical areas. A county with a large urban region is classified as metropolitan, although it may also house extremely rural regions devoid of the services available in the metro region. Additionally, residents on the border of counties may have access to goods and services in the adjoining county that are closer to them than those within their county of residence [14]. Some codes try to account for commuting factors, but they are ultimately based on the county-level OMB designation of rurality, so overbounding and underbounding are common [7, 11, 15]. These limitations must be considered when applying any of these taxonomies to rural health research.

### 2.1. Measures of Access to Health Services

 In addition to measures of rurality, health research and policy commonly use several specific measures of access to health services to designate underserved areas. These designations are based on provider-to-population ratios, not necessarily population density and were developed to highlight gaps in service availability and delivery, equivalent to access to care. While the underserved designations apply equally to rural and urban populations, these designations most often coincide with rural areas, while metropolitan areas occasionally have smaller designated areas or subpopulations within them. Two designations are mostly used—the Health Professional Shortage Area (HPSA) and the Medically Underserved Area (MUA); both designations are managed by the US Health Resources and Services Administration and are used to allow certain communities to be eligible for specific types of funding to improve their access to services.

The primary care Health Professional Shortage Area designation is based on the ratio of primary care providers to the population. If the ratio is less than 1 : 3 per 500 then the region is designated as a shortage area; the mental health and dental Health Professional Shortage Area designations have their own unique provider ratios. Once a Health Professional Shortage Area is identified, it is scored based on poverty line, Infant Health Index, and distance to the healthcare. Regions that do not automatically qualify for Health Professional Shortage Area status can appeal or be granted such status if other significant health needs are demonstrated. The Health Professional Shortage Area system varies from either the Urban Influence Codes or Rural Urban Continuum Codes in that it is not entirely county based. Because the criteria for Health Professional Shortage Area designation are calculated as a ratio, population density itself is not considered. A shortage area can be geographic, a population group, or a facility, with each area defined by unique guidelines. The Health Professional Shortage Area designation brings with it eligibility to receive certain federal benefits, training, and loan repayment [16, 17].

Medically Underserved Areas use Index of Medical Underservice (IMU) which measures infant mortality, percent of population below the poverty line, percent of the population 65 or older, and the number of primary care providers per 1,000 people. Like the Health Professional Shortage Area, the Medically Underserved Area designation is not based solely on population density and can be given to regions that do not exactly fit the criteria if they can document extraordinary barriers to health services and the designation is advocated by state health officials [16]. Health Professional Shortage Area and Medically Underserved Area designations go beyond population size within a geographic boundary to account for additional factors—many of which are common in rural areas—affecting access to care.

## 3. Study Aims

 Clearly, although the different taxonomies for defining rurality share certain characteristics, they each hinge on specific aspects of ruralness while not including others. The designations of underserved are based on relatively objective measures, and the vast majority of rural communities fall into one or more of the underserved categories. Extensive research indicates that rural health disparities are pervasive—particularly around access to care. This raises the question, to what degree do the different designations of rural and underserved coincide and offer similar conclusions when applied to the same research question regarding access to care? Given the previous descriptions of rural designations provided previously, the remainder of the paper will utilize data collected through an eight-county health assessment to

1. identify the extent to which various designations of rurality and medically underserved overlap in an eight-county region of Central Texas that includes variation in population density and other characteristics;
2. examine the key characteristics of the cluster profiles generated based on distance traveled to obtain health services; and
3. describe unique and common participant and cluster profile characteristics by association with each of the 4 rural designation classifications.

## 4. Materials and Method

### 4.1. Participants and Procedures

 Data were collected in 2010 as part of a regional eight-county health assessment of the Brazos Valley in Central Texas. The survey was conducted by the Center for Community Health Development at the Texas A&M School of Rural Public Health and was intended to assist local communities in identifying and prioritizing health problems. Results of this survey, conducted approximately every 4 years, are used by the Brazos Valley Health Partnership as part of their planning for community health action. The assessment utilized random-digit dialing to obtain a population-based sample of the noninstitutionalized civilian population. Sampling was stratified by county to ensure adequate representation of counties in the region.

Further randomization within each household was achieved using the next-birthday method. That is, when making recruiting phone calls, investigators asked to speak with the adult resident present in the household who had the birthday that would next occur. That resident was then informed of the survey purpose and recruited to participate in the assessment. Of those reached by phone, 51.9% agreed to participate and received a paper survey by mail. Two reminder postcards were sent at 2-week intervals following mail-out of the survey packet. Of those who were sent surveys, 62.1% (*N* = 3,946) returned completed surveys (i.e., overall response rate = 32.2%). Institutional Review Board approval was obtained at Texas A&M University.

### 4.2. Instrument

 Participants were surveyed using a mailed community assessment instrument that asked questions about the respondent's health, lifestyle behaviors, health care access, neighborhood factors, and personal characteristics. The instrument included Likert-type scales, checklists, and close-ended and open-ended response formats. Participants took approximately 45 minutes to complete the questionnaire.

### 4.3. Data and Measures

#### 4.3.1. Dependent Variables

Rurality and medically underserved designations were used as the dependent variables for this study (i.e., Urban Influence Codes, Rural Urban Commuting Area, National Center for Health Statistics Urban-Rural Classification (NCHS), Medically Underserved Area, Health Professional Shortage Area, Frontier). All 8 counties were classified using the criteria specific to each of the designations separately. Counties were coded into dichotomous categories for each designation based on the respective categorization recommendation (i.e., metropolitan/rural; Medically Underserved Area/not Medically Underserved Area; Health Professional Shortage Area/not Health Professional Shortage Area; frontier/not frontier). County-level assignments were identical for Urban Influence Codes, Rural Urban Commuting Area codes, and National Center for Health Statistics Urban-Rural Classification codes, thus these designations were combined as 1 category for study analyses (i.e., metro, nonmetro). Medically Underserved Areas, Health Professional Shortage Areas, and Frontier were categorized as either “not designated” or “designated.” All dichotomized designations were commonly coded as 0 and 1, whereas the score of 1 consistently refers to the rural or underserved designation (i.e., nonmetro, medically underserved areas, health provider shortage areas, frontier areas).

#### 4.3.2. Distance Traveled to Medical Services

The survey gathered self-reported distance driven as a measure for access to healthcare. Regarding distance traveled, Fortney and colleagues noted that GIS-based systems can accurately assess access to healthcare [18], and Witlox and colleagues validated that self-reported distance is a reliable measure of distance travelled which is comparable to GIS-computed distance [19]. With that in mind, self-reported measures of distance to healthcare should be viewed as a reasonably accurate and cost-effective manner of measuring healthcare access.

Participants were asked to self-report the distance they traveled from their home to obtain healthcare services. Specifically, participants were asked how many miles they traveled from home to (1) their medical care facility, (2) their dental care facility, and (3) retrieve prescription medications. These variables were scored continuously and used in the clustering process described later.

#### 4.3.3. Health Indicators

Body mass index (BMI) was calculated from participants' self-reported height (in feet and inches) and weight (in pounds), which were converted to meters and kilograms, respectively. BMI levels were calculated by dividing weight by height and rounded to the nearest tenth [20], and BMI categories were then created as follows: normal weight (BMI = 18.5 to 24.9 kg/m^2^), overweight (BMI = 25 to 29.9 kg/m^2^), and obese (BMI ≥ 30 kg/m^2^) (WHO, 1998). Participants' self-reported the number of diagnosed chronic condition types, which were recorded as a continuous variable ranging from 0 to 5 (i.e., cardiovascular, diabetes, cancer, respiratory disease, and other conditions).

#### 4.3.4. Personal Characteristics

Personal characteristics of the participants included age, collected as a continuous variable then recoded into meaningful age groups (i.e., 18 to 40 years, 41 to 64 years, 65+ years), sex (i.e., male, female), race/ethnicity (i.e., non-Hispanic white, African American, Hispanic), and highest education level achieved (i.e., less than high school, graduate high school, and more than high school).

### 4.4. Clustering Processes

#### 4.4.1. Selection of Cluster Variables

Consistent with the research questions of our study, we selected variables for cluster analysis that were indicative of the ability of respondents to reach healthcare services. Respondents were asked how far they traveled (in number of miles) to get medical care, dental care, and prescriptions. We only considered respondents who answered these questions in the context of starting these travels from their home (*n* = 2,375) versus their place of work or another location. These three variables (distance to medical care, dental care, and prescriptions) were the basis for creating the clusters.

#### 4.4.2. Transformation of Variables

The standard deviation among the three variables chosen for cluster analysis was substantial. Most respondents indicated traveling only a short distance, but other respondents indicated traveling substantial distances. To avoid biasing the study toward respondents living relatively close to medical services, we used a log transformation of the distance variables to achieve a normalized distribution of the variables. This process allowed for a better representation of differences between respondents and led to more cohesive clusters.

#### 4.4.3. Missing Data and Outliers

Based on variable selection for clustering as well as the log transformation, the number of observations was reduced based on missing data (*n* = 367). These observations were listwise deleted by Stata version 11 (StataCorp, College Station, TX), resulting in a sample of 1,958 observations for clustering.

#### 4.4.4. Cluster Analytic Methods and Proximity Measures

K-means clustering was selected as the clustering method since it is uniquely designed for nonhierarchical data partitioning. Because the underlying variables of the cluster analysis (distance) were expressed in mileage, Euclidean distance was used as the proximity function because of its utility in continuous variable analysis [21].

#### 4.4.5. Number of Clusters

Determining the number of clusters to extract was a key consideration. We created 10 different cluster models to examine the change in both the within-group and between-group sum of squared errors (SSE) as additional clusters were added. Based on guidance from established literature [22, 23], the design of our study, and our desire to achieve the most parsimonious results, the model minimizing within-group SSE was selected as the final cluster model.

### 4.5. Statistical Analyses

 We assessed the proportional overlap of study participants residing within each designation using Kendall tau rank correlation coefficients and a series of chi-squared tests. Personal characteristics, health indicators, distance traveled to healthcare services, and rurality designations were described by cluster profiles. Differences between cluster profiles were identified using chi-squared for categorical variables and tests and analyses of variance (*F*-tests) for continuous variables. Then, four independent binary logistic regression analyses were performed to assess factors associated with residing in “more rural” areas (as deemed by each designation, resp.).

## 5. Results

**Figure 1:**
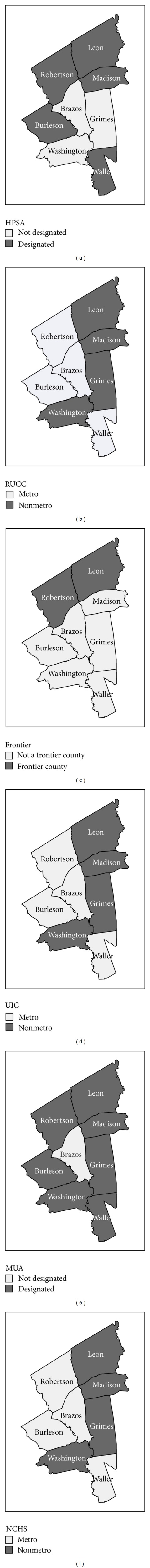
Rural designation by county.

 After reviewing the various definitions of rurality, an initial aim was to identify the extent to which various designations of rurality and medically underserved overlap in an eight-county region of Central Texas that includes variation in population density, and other characteristics. As illustrated in Figure 1, measures of rurality and underserved differed among the eight counties in this Central Texas region based on the type of designation used. Using six rurality-related designation criteria to categorize the Brazos Valley region of Texas resulted in four configurations of these eight counties. One county consistently remained “rural/frontier,” and one county consistently remained “urban” regardless of which criteria were applied, but substantial variability was observed among the remaining counties. Additional variations were seen in the underserved designations.

Designation comparisons by participant residence are provided in Table 1. All participants residing in counties designated as frontier also resided in Medically Underserved Areas and Health Professional Shortage Areas. Of the participants residing in counties not designated as frontier, 58.4% resided in counties designated as Medically Underserved Areas and 23.8% resided in counties designated as Health Professional Shortage Areas. Participants residing in Health Professional Shortage Areas also resided in Medically Underserved Areas; however, 45.4% of those residing in nondesignated Health Professional Shortage Areas were considered to reside in designated Medically Underserved Areas. Proportions of participants residing in both “rural” and “urban” designations were observed when comparing Urban Influence Codes to Health Professional Shortage Areas and Frontier areas, respectively.

Another study aim was to examine the key characteristics of the cluster profiles generated based on distance traveled to obtain health services.  Table 2 describes sample characteristics by cluster profiles. Cluster profiles are organized from smallest to largest based on the total number of miles traveled to medical services. Approximately 46% of sample participants were aged 41 to 64 years and 61.7% were aged 65 years and older. The majority of participants was female (70.1%), non-Hispanic white (81.1%), and had more than a high school education (57.2%). About 35% of participants were classified as overweight and 34.7% as obese. On average, participants self-reported having 1.32 (±1.13) chronic conditions and driving a total of 52.49 (±48.13) miles to healthcare services. When comparing sample characteristics by cluster, significant differences were observed by age group (*χ* ^2^ = 57.80, *P* < 0.001) and race/ethnicity (*χ* ^2^ = 33.59, *P* < 0.001). Significant differences were observed based on distances traveled to obtain healthcare services, whereas the distances traveled were greater as the cluster number increased. Further, significant differences based on rurality designation of participants' residence significantly differed, whereas the proportion of “rural” designations was significantly larger as the cluster number increased.

The final study aim was to describe unique and common participant and cluster profile characteristics by association with each of the 4 rural designation classifications.  Table 3 contains results from 4 independent binary logistic regression analyses using the “urban” category of each designation as the referent group for each model, respectively. Participants residing in “rural” Urban Influence Codes, Rural Urban Commuting Areas, and National Center for Health Statistics Urban-Rural Classification areas were significantly more likely to be age 65 years (OR = 1.35, *P* = 0.009) and older; rural residents were also more likely to be African American (OR = 2.64, *P* < 0.001). Participants residing in designated Medically Underserved Areas were significantly more likely to be age 65 years and older (OR = 1.69, *P* < 0.001), African American (OR = 3.71, *P* < 0.001), Hispanic (OR = 2.14, *P* = 0.006), and have more than a high school education (OR = 1.45, *P* = 0.005). Participants residing in designated HSPA were significantly less likely to be age 41 to 64 years (OR = 0.66, *P* = 0.046). Those who were African American (OR = 1.60, *P* = 0.030), overweight (OR = 1.39, *P* = 0.019), obese (OR = 1.33, *P* = 0.031), and those with more than a high school education (OR = 1.55, *P* < 0.001) were significantly more likely to reside in designated Health Professional Shortage Areas. Participants who were African American (OR = 2.19, *P* = 0.008), Hispanic (OR = 2.49, *P* = 0.010), obese (OR = 1.58, *P* = 0.003), and those with more than a high school education (OR = 1.66, *P* < 0.001) were significantly more likely to reside in frontier counties. In all regression models, relative to the cluster profile representing those traveling the shortest overall distance to obtain healthcare services (i.e., cluster 1), those traveling farther distances (i.e., clusters 2 through 5) were significantly more likely to reside in “rural” designated areas.

## 6. Discussion

 While the complexities of rurality-related designations have been identified and compared by other researchers [7], fewer studies have directly assessed these designations as they relate to distance traveled to common medical services. Although travel distance required to obtain healthcare services disproportionately impacts rural-residing populations' utilization of preventive and routine healthcare [6, 24], distance traveled is not consistently observed as a limiting factor for healthcare services [25]. Distance traveled to obtain services is an important consideration and characteristic of traditionally defined rural areas; however, research findings and associated implications based on greater distances traveled should be contextualized by rural designation and the types of services being acquired.

The current study offers valuable insights for disparities research that examines relationships relevant to rural areas and access to care. Given that the taxonomies for designating certain areas as rural or as provider shortage areas are widely used in public health research, the results of this study highlight discrepancies among conclusions that may be drawn based upon the application of the different definitions of rurality when applied to health data. These discrepancies point to four important conclusions.

First, various aspects of a community factor into whether it is considered rural or underserved, which transcends its geography, population density, or distance to services. The current designations focus on a few key characteristics but cannot fully capture all of the aspects that collectively define rurality or access to care. The designations of rural and medically underserved examined in this study, while useful for specific purposes, lack key components to make the meanings they convey interchangeable. Rural designations focus on geography and population density, while underserved designations focus on provider to population ratios, infant mortality, percent of population below poverty level, and percent of population over 65 years of age. As seen in this study, the same area can be classified in multiple ways depending upon the designation criteria applied. Of the eight counties examined, only two were consistently designated regardless of the criteria used. More often, we found significant variation in whether counties were designated as rural or frontier, as Health Professional Shortage Areas, or Medically Underserved Areas. The application of different designations results in differing descriptions for the same county; this reinforces the conclusion that it is difficult to generalize health risks and health disparities among rural populations, even within the same geographic region.

Second, it can be argued that there are no “rural” designations that are designed specifically for applications in health research. Consequently, public health researchers are forced to utilize taxonomies developed by agencies focused on other issues when parceling out segments of the population to strengthen our understanding of health issues and disparities. Although this practice is very common, it is not without problems. The issues with using other disciplines' designations in population health data are typically acknowledged in one sentence in the limitations section of a research article and not further explored. The current study adds to what we know about those limitations that may need to be addressed more thoroughly.

Third, repeating the same study with the same data but applying different definitions of rural and medically underserved may result in very different conclusions. As illustrated in the current study, applying six different criteria yielded four divergent models of rurality and underserved-ness for the study region, with only two counties consistently classified across all 4 models. When analyzed together, both rurality and underserved designations are likely to coincide when defining only two types of communities: metropolitan and frontier. Metropolitan communities have a large population density, shorter distances to care, and are less likely to be designated as Health Professional Shortage Areas or Medically Underserved Areas. On the other hand, frontier communities are nonmetropolitan communities with small population densities and longer distances to care and are likely to also be designated as Health Professional Shortage Areas and Medically Underserved Areas. However, how do the designations of rurality and medically underserved define those areas that are neither metropolitan nor frontier? Based on current designations, these communities are defined by what they are not rather than what they are. These are the areas in which applying different designations to the same data yields different conclusions. Thus, generalizing to the rural communities is much more difficult that to urban and frontier communities.

Finally and perhaps most importantly, given that research employing these various definitions of rurality is used to inform health policy that ultimately affects people residing in rural areas, we must be cognizant of the implications this may have. Rural populations often face challenges in the implementation of policies that are developed for urban communities with greater population and resource concentration. When researchers specifically examining rural populations recommend policy based on conclusions of research using one taxonomy or another, it is critical to know the assumptions and limitations of that designation, as well as how using a different established designation may change those conclusions. These differences may greatly affect the resulting policy development.

This study also has policy implications. While the designations included in this study inconsistently overlap, it is important to recognize that various funding agencies as well as governmental and community-based organizations utilize these different calculations and computations to target populations in need of resources, programs, and services. As such, the designation used to identify target populations may impact decisions about the types of intervention strategies selected to address health issues in that area or ways in which success of the efforts is measured/determined. This study highlights the importance of researchers and community-based organizations to investigate designation categories in which relevant and allied agencies are used to allocate funding or services, so they may better understand the missions of these organizations and their basis for prioritizing certain designations over others. Researchers and organizations can subsequently align their efforts and proposals to ensure compatibility and avoid the potential for service gaps associated with area misclassification.

Although this study contributes with an important perspective to the existing literature, several limitations must be acknowledged. The primary limitation of this study is that the population survey was conducted in eight contiguous counties in Central Texas; thus, the data only represent one region, and data from other regions may indicate a variety of other discrepancies. Additional research should replicate this cluster analysis to determine what those might be. In addition, while the sample size was sufficient for the analysis, a greater sample with more variability would undoubtedly strengthen the conclusions drawn. Finally, the sample did not include a comparison area of a mega-urban population; it is unclear what those data may have suggested related to the application of Health Professional Shortage Area and Medically Underserved Area criteria and access to care measures. Future research should aim to incorporate such populations into their samples.

## 7. Conclusion

In conclusion, it is important to highlight that definitions of rurality were not developed with health factors in mind, thus they were not created specifically to be used in health research. When applying them without acknowledging their original purpose and thus recognizing the subsequent limitations, misleading conclusions may be drawn from certain types of data analysis. However, it is not our contention that any of these taxonomies be abandoned in health studies. Rather, these designations should be used with caution, and researchers and policymakers must respect differences across various categorizations and acknowledge their limitations in application and interpretation.

## Figures and Tables

**Table 1:** Distribution comparison by rural designation.

	MUA		HPSA		Frontier	
	Not designated (*n* = 619)	Designated (*n* = 1339)	Not designated (*n* = 1133)	Designated (*n* = 825)	Not designated (*n* = 1487)	Designated (*n* = 471)
UIC						
Metro (*n* = 1030)	100.0%	30.7%	54.6%	49.8%	54.7%	46.1%
Nonmetro (*n* = 928)	0.0%	69.3%	45.4%	50.2%	45.3%	53.9%
	*χ* ^2^ = 815.52 *P* < 0.001		*χ* ^2^ = 4.44 *P* = 0.035		*χ* ^2^ = 10.62 *P* = 0.001	
	*τ* = 0.56 *P* < 0.001		*τ* = 0.24 *P* < 0.001		*τ* = 0.25 *P* < 0.001	

MUA						
Not designated			54.6%	0.0%	41.6%	0.0%
Designated			45.4%	100.0%	58.4%	100.0%
			*χ* ^2^ = 659.09 *P* < 0.001		*χ* ^2^ = 286.70 *P* < 0.001	
			*τ* = 0.58 *P* < 0.001		*τ* = 0.38 *P* < 0.001	

HPSA						
Not designated					76.2%	0.0%
Designated					23.8%	100.0%
					*χ* ^2^ = 851.72 *P* < 0.001	
					*τ* = 0.66 *P* < 0.001	

UIC: Urban Influence Codes.

MUA: Medically Underserved Area.

HPSA: Health Professional Shortage Area.

**Table 2:** Sample characteristics by cluster.

	Cluster 1 (*n* = 188)	Cluster 2 (*n* = 348)	Cluster 3 (*n* = 450)	Cluster 4 (*n* = 468)	Cluster 5 (*n* = 504)	Total (*n* = 1958)	*χ* ^2^ or *t*	*P*
Age							57.796	<0.001
18–40 years	7.4%	14.4%	14.3%	13.1%	10.5%	12.4%		
41–64 years	30.9%	37.6%	49.1%	49.5%	52.3%	46.2%		
65+ years	61.7%	48.0%	36.6%	37.4%	37.3%	41.4%		
Sex							4.374	0.358
Male	29.3%	34.2%	28.0%	30.4%	28.6%	29.9%		
Female	70.7%	65.8%	72.0%	69.6%	71.4%	70.1%		
Education							13.737	0.089
Less than high school	7.0%	5.2%	5.3%	6.2%	3.4%	5.2%		
High school graduate	33.7%	31.9%	40.0%	39.7%	38.8%	37.6%		
More than high school	59.4%	62.9%	54.6%	54.1%	57.8%	57.2%		
Race/ethnicity							33.592	<0.001
Non-Hispanic white	79.8%	74.9%	76.7%	84.2%	87.1%	81.1%		
African American	12.0%	11.4%	10.5%	6.0%	6.1%	8.6%		
Hispanic	8.2%	13.7%	12.8%	9.9%	6.9%	10.3%		
Body mass index							13.557	0.094
Normal weight	28.0%	30.7%	27.7%	33.9%	28.2%	29.9%		
Overweight	41.8%	35.8%	34.6%	35.7%	33.3%	35.4%		
Obese	30.2%	33.4%	37.8%	30.4%	38.6%	34.7%		
Number of chronic conditions	1.42 (±1.08)	1.32 (±1.17)	1.32 (±1.10)	1.28 (±1.11)	1.30 (±1.15)	1.32 (±1.13)	0.536	0.709
Distance traveled to (in miles)								
Medical care facility	3.59 (±6.29)	3.33 (±1.96)	14.32 (±14.96)	23.29 (±17.05)	45.01 (±26.70)	21.41 (±23.51)	388.041	<0.001
Dental care facility	1.24 (±0.58)	6.39 (±11.01)	14.26 (±17.42)	15.62 (±8.00)	42.70 (±25.89)	19.27 (±22.13)	371.269	<0.001
Retrieve prescription medications	1.24 (±0.83)	2.50 (±1.69)	4.43 (±2.27)	14.27 (±8.26)	26.45 (±17.75)	11.81 (±13.94)	472.017	<0.001
Total	6.07 (±6.14)	12.21 (±10.31)	33.02 (±21.35)	53.18 (±17.59)	114.15 (±46.52)	52.49 (±48.13)	1011.251	<0.001
UIC								
Metro	12.5%	25.5%	23.9%	20.2%	17.9%	52.6%	158.623	<0.001
Nonmetro	6.4%	9.2%	22.0%	28.0%	34.5%	47.4%		
MUA								
Not designated	17.1%	37.0%	30.5%	14.1%	1.3%	31.6%	510.874	<0.001
Designated	6.1%	8.9%	19.5%	28.5%	37.0%	68.4%		
HPSA								
Not designated	13.2%	25.9%	28.1%	20.4%	12.4%	57.9%	365.209	<0.001
Designated	4.7%	6.5%	16.0%	28.7%	44.0%	42.1%		
Frontier								
Not designated	11.9%	21.8%	26.1%	22.9%	17.3%	75.9%	290.413	<0.001
Designated	2.3%	5.1%	13.2%	27.0%	52.4%	24.1%		

UIC: Urban Influence Codes.

MUA: Medically Underserved Area.

HPSA: Health Professional Shortage Area.

**Table 3:** Factors associated with rurality designation (*n* = 1790).

	UIC				MUA				HPSA				Frontier			
			95% CI				95% CI				95% CI				95% CI	
	OR	*P*	Lower	Upper	OR	*P*	Lower	Upper	OR	*P*	Lower	Upper	OR	*P*	Lower	Upper
Age: 18–40 years	—	—	—	—	—	—	—	—	—	—	—	—	—	—	—	—
Age: 41–64 years	1.23	0.284	0.84	1.79	1.23	0.353	0.80	1.90	0.66	0.046	0.44	0.99	0.67	0.119	0.41	1.11
Age: 65+ years	1.35	0.009	1.08	1.68	1.69	<0.001	1.30	2.22	0.84	0.144	0.66	1.06	0.81	0.140	0.62	1.07
Male	—	—	—	—	—	—	—	—	—	—	—	—	—	—	—	—
Female	1.18	0.143	0.95	1.47	1.12	0.405	0.86	1.45	0.88	0.294	0.70	1.12	0.94	0.665	0.72	1.23
Education: less than high School	—	—	—	—	—	—	—	—	—	—	—	—	—	—	—	—
Education: high school graduate	1.28	0.353	0.76	2.15	1.44	0.228	0.80	2.60	1.04	0.889	0.59	1.83	1.33	0.406	0.68	2.60
Education: more than high School	1.16	0.156	0.94	1.43	1.45	0.005	1.12	1.88	1.55	<0.001	1.24	1.93	1.66	<0.001	1.29	2.13
Non-Hispanic white	—	—	—	—	—	—	—	—	—	—	—	—	—	—	—	—
African American	2.64	<0.001	1.77	3.95	3.71	<0.001	2.38	5.77	1.60	0.030	1.05	2.45	2.19	0.008	1.23	3.91
Hispanic	1.43	0.162	0.87	2.37	2.14	0.006	1.24	3.70	1.39	0.229	0.81	2.38	2.49	0.010	1.25	4.97
BMI: normal weight	—	—	—	—	—	—	—	—	—	—	—	—	—	—	—	—
BMI: overweight	1.00	0.998	0.77	1.30	1.18	0.302	0.86	1.61	1.39	0.019	1.06	1.84	1.24	0.188	0.90	1.72
BMI: obese	1.09	0.499	0.85	1.39	1.17	0.283	0.88	1.57	1.33	0.031	1.03	1.73	1.58	0.003	1.17	2.13
Number of chronic conditions	1.04	0.412	0.94	1.15	1.04	0.557	0.92	1.17	1.03	0.529	0.93	1.15	1.08	0.225	0.96	1.21
Health services travel: Cluster 1	—	—	—	—	—	—	—	—	—	—	—	—	—	—	—	—
Health services travel: Cluster 2	0.48	<0.001	0.36	0.63	0.03	<0.001	0.01	0.05	0.17	<0.001	0.13	0.23	0.16	<0.001	0.11	0.22
Health services travel: Cluster 3	0.70	0.012	0.53	0.93	0.08	<0.001	0.04	0.17	0.39	<0.001	0.30	0.52	0.36	<0.001	0.27	0.48
Health services travel: Cluster 4	0.28	<0.001	0.19	0.41	0.02	<0.001	0.01	0.03	0.10	<0.001	0.07	0.16	0.06	<0.001	0.03	0.11
Health services travel: Cluster 5	0.21	<0.001	0.15	0.28	0.01	<0.001	0.01	0.02	0.07	<0.001	0.05	0.11	0.07	<0.001	0.04	0.11

	*Referent group: metro designation				*Referent group: non-underserved area				*Referent group: nonshortage area				*Referent group: nonfrontier designation			
	Nagelkerke *R* ^2^ = 0.137				Nagelkerke *R* ^2^ = 0.392				Nagelkerke *R* ^2^ = 0.252				Nagelkerke *R* ^2^ = 0.248			

UIC: Urban Influence Codes.

MUA: Medically Underserved Area.

HPSA: Health Professional Shortage Area.
